# Mental Health Services for Individuals with High Functioning Autism Spectrum Disorder

**DOI:** 10.1155/2014/502420

**Published:** 2014-09-03

**Authors:** Johanna K. Lake, Andrea Perry, Yona Lunsky

**Affiliations:** ^1^Dual Diagnosis Service, Centre for Addiction and Mental Health, 501 Queen Street West, Toronto, ON, Canada M5V 2B4; ^2^Department of Psychiatry, University of Toronto, 250 College Street, 8th Floor, Toronto, ON, Canada M5T 1R8

## Abstract

Adolescents and adults with an autism spectrum disorder (ASD) who do not have an intellectual impairment or disability (ID), described here as individuals with high-functioning autism spectrum disorder (HFASD), represent a complex and underserved psychiatric population. While there is an emerging literature on the mental health needs of children with ASD with normal intelligence, we know less about these issues in adults. Of the few studies of adolescents and adults with HFASD completed to date, findings suggest that they face a multitude of cooccurring psychiatric (e.g., anxiety, depression), psychosocial, and functional issues, all of which occur in addition to their ASD symptomatology. Despite this, traditional mental health services and supports are falling short of meeting the needs of these adults. This review highlights the service needs and the corresponding gaps in care for this population. It also provides an overview of the literature on psychiatric risk factors, identifies areas requiring further study, and makes recommendations for how existing mental health services could include adults with HFASD.

## 1. Introduction

When it comes to supporting the needs of adolescents and adults with an autism spectrum disorder who do not have intellectual impairment or disability, described here as individuals with high-functioning autism spectrum disorder (HFASD), our mental health system is in crisis. We know that rates of mental health issues are high in this population; yet accessing services to address these symptoms remains difficult. Addressing the mental health and intervention needs of adolescents and adults with ASD is a recognized priority in Canada [1–3], the United States [4], and the United Kingdom [5–7], but most individuals with HFASD continue to encounter significant difficulties in social relationships, employment, and communication. These issues are further magnified in developing countries where there is an emerging literature on the diagnosis and treatment of ASD, but where the mental health needs of individuals with ASD often exceed available resources, and where limited training, policy, and mental health programs exist [8, 9].

The Centers for Disease Control and Prevention (CDC) in the United States estimate that 1 in 68 children have an autism spectrum disorder (ASD) and almost half (46%) function in the average or above average range of intellectual ability (IQ > 85) [10]. Previously defined as* Asperger syndrome* (AS) in the tenth revision of the International Statistical Classification of Diseases and Related Health Problems (ICD-10; [11]) and the fourth revised edition of the Diagnostic and Statistical Manual of Mental Disorders (DSM-IV-TR; [12]), individuals with HFASD are characterized by deficits in reciprocal social interaction and patterns of behaviour or interests in the absence of clinically significant delays in receptive language or cognitive development. In the DSM-5 [13], these individuals are now characterized as individuals with ASD who do not have an intellectual impairment. For the purpose of this review, we broadly refer to all individuals with AS or high-functioning autism as HFASD, even if some studies used different diagnostic terms. Although we know that children with ASD grow up to be adults with ASD, there are fewer mental health services available for adults with ASD, particularly for individuals without an ID. Despite improved recognition of individuals functioning at all levels across the spectrum, access to care for individuals with HFASD and research on this population has not kept pace with their needs.

As in the case with most developmental disorders, ASD symptomatology changes with age. While there is a significant body of literature on symptoms, service use, and interventions for children with ASD, there are fewer studies that have investigated these issues in adults [14, 15]. This is well articulated in the United Kingdom National Autistic Society document, “Ignored or ineligible? The reality for adults with autism spectrum disorders,” where findings of 1200 adults with ASD demonstrated that many parents of these adults were confused about which statutory agency was responsible for their child's care and that the responsibility for funding and providing care and support often fell between agencies [16]. The issue of accessing mental health services is further complicated for adults with HFASD because community mental health agencies are not typically inclusive of individuals with ASD. The impact of this scenario is a lack of recognition among community mental health programs that this is a population needing their care for whom they are well equipped to support. Poor recognition occurs for a number of different reasons including restrictive intake criteria, misdiagnosis, limited knowledge or awareness of ASD, clinicians who lack confidence or exposure in caring for this population, and the notion that another service provider will provide this care.

Generally, individuals with HFASD have been unable to access developmental disability services because of the absence of significant cognitive impairments, leaving their needs to be addressed in community mental health settings. However, some adolescents and adults with HFASD, because of their ASD diagnosis, are excluded from community mental health services, leaving them grossly underserved. The purpose of this review, therefore, is to discuss some of the most common issues facing adolescents and adults with HFASD and to describe some of the interventions developed to support this population. In doing so, we also describe areas for future study, identify significant challenges and barriers to accessing mental health services, and highlight opportunities to address these barriers within existing community mental health systems and services. It is important to note that the interventions proposed in this review are services and supports which already form the foundation of many existing community mental health programs.

## 2. Mental Health Issues in HFASD

Comorbid psychiatric disorders are well documented in individuals with ASD across the lifespan [17–24]. Compared to studies of psychiatric comorbidity in individuals with ASD generally, research on cooccurring psychiatric disorders in adults and teens with HFASD, specifically, has been slower to progress. Most evidence comes from studies of youth (children and early adolescents), with fewer studies focusing on older adolescents and adults. Of these studies, findings suggest that a very high proportion of adults and teens with HFASD present with comorbid psychiatric disorders, particularly depression and anxiety [14, 17, 22, 25–29]. By contrast, studies of youth with HFASD tend to report higher rates of behavior and attention problems [27, 30, 31].

In a community and clinic-based study of adolescents with HFASD, results identified a prevalence rate of 74% for one or more comorbid psychiatric disorders, with 44% of individuals diagnosed with behavioural disorders and 44% diagnosed with anxiety disorders [27]. In another study, Ghaziuddin and Greden [32] identified that 65% of patients with HFASD also presented with symptoms of an additional psychiatric disorder. Children with HFASD were most likely to have a comorbid diagnosis of ADHD, while depression was the most common diagnosis in adolescents and adults. Similar findings were reported for rates of bipolar disorder in an outpatient clinic of 54 young adults with HFASD, where 36% of patients were diagnosed with a mood disorder [28]. More recently, in a study examining psychiatric comorbidity in young adults with AS, 70% had experienced at least one episode of major depression, 50% had suffered from recurrent depressive episodes, and 50% met criteria for an anxiety disorder [26]. Similar findings were reported in a study of adolescents with HFASD, where individuals with HFASD reported more psychiatric symptoms, particularly withdrawn, anxious/depressed, and social problems, compared to controls [25].

In summary, although research on adults and teens with HFASD is limited, studies to date suggest that the cooccurrence of psychiatric disorders in this population is remarkably high. Specifically, higher functioning adolescents and adults appear to be at heightened risk for developing depression and anxiety [33–35]. Despite these findings, several studies reported that comorbid psychiatric disorders and even the diagnosis of ASD itself often go unrecognized among adolescents and adults with HFASD seeking psychological or psychiatric care [28, 36]. This information is particularly noteworthy in the context of autism-related sociocommunicative deficits, which, when combined with psychiatric symptoms, can be quite debilitating in this population.

## 3. Long-Term Outcomes in HFASD

### 3.1. Challenges to Functional Autonomy and Quality of Life

A handful of longitudinal studies have demonstrated the many challenges youth with HFASD encounter once they become adults. Despite having intact cognitive skills, the large majority of adults and adolescents with HFASD continue to struggle with finding meaningful and gainful employment, as well as some degree of autonomy and independence in their lives [14]. For example, in a study examining outcomes of 19 men with HFASD, half had been educated in a special autism class, 5% had obtained independent employment, 16% worked in sheltered or voluntary jobs, and 11% were in full time education. In terms of housing, 50% of these adults lived in residential or supported accommodation, 40% with their parents, and 5% in a long-term facility for individuals with developmental delay [37]. Critically, only 10% of these high-functioning individuals lived independently.

Clearly, despite having normal intelligence, adults with HFASD present with significant delays in their adaptive skills. Most of these individuals continue to live at home with their parents and if employed obtain jobs in low level, poorly paid, sheltered or supported employment. It is hoped that in the future interventions will continue to improve for this population, with greater attention to social outcomes in school age youth and increased supports at the college level (e.g., mentorship programs). For example, findings of a recent case study of students with HFASD and their university coordinators demonstrated the benefits of explicit, organized routines and structures and of the importance of support systems (e.g., relatives) and services tailored to the individuals' needs [38].

### 3.2. Persistence of Social Challenges

Deficits in social functioning are a central feature of ASD regardless of level of intellectual functioning. Social skills deficits in this population do not remit with age and may in fact increase during adolescence and adulthood [39, 40]. Studies of children with HFASD demonstrate that despite having intact verbal skills, these individuals often fail to utilize language appropriately in social interactions, particularly in understanding nonliteral language, humor, and irony [41]. Individuals with HFASD may also be more acutely aware of the difficulties they encounter during social interactions compared to individuals with ASD with an ID and therefore appear to be at greater risk for developing depression, low self-esteem, and anxiety, when faced with peer rejection or bullying [42]. In a comparison study of adolescents and adults with HFASD and controls without ASD, individuals with HFASD reported significantly lower scores on measures of friendship [43]. Similarly, in another study, this time of 19 men with HFASD, only three reported close friendships and none had married [37]. Consistent findings were reported in several more recent studies of adolescents and adults with HFASD [44–46].

### 3.3. Challenges Accessing Services

Youth with ASD report significant difficulties accessing healthcare services, particularly comprehensive health services [47, 48]. Part of the reason for this difficulty stems from service providers feeling ill equipped to work with individuals with ASD, particularly individuals with comorbid mental health issues [1]. Challenges accessing services can often precipitate crises for individuals with ASD, leading families to be distrustful of healthcare services and service providers [49]. To date, no longitudinal research has examined whether these problems increase or decrease into adulthood.

In Ontario, as well as other parts of Canada and the world, the majority of services for adolescents and adults with ASD are designed for people with an intellectual disability [1]. Since these individuals do not meet criteria for an ID, adolescents and adults with HFASD have access to significantly fewer programs than adolescents and adults with other types of developmental disabilities. All of this occurs during a particularly difficult time of transition whereby social demands start to increase and educational and occupational structures are decreased [1, 50]. Not surprisingly then, a consistent theme for parents of individuals with HFASD is the fear that their child will fall through the cracks when transitioning from child to adult services [51]. Similar concerns have been voiced by the individuals with HFASD themselves, who describe how their needs are rarely recognized and the programs and services available are not designed for people with ASD in mind [52]. From the perspective of community mental health agencies, many services do not want to accept clients with ASD who have comorbid psychiatric or behaviour disorders because they do not feel equipped to manage their care.

While the majority of outcome studies showed improvement in ASD symptoms over time, most individuals with HFASD continue to encounter significant difficulties in social relationships, employment, and communication. Additionally, parents of these teens and adults report a number of challenges and concerns related to future independence and availability of appropriate services and supports.

## 4. Mental Health Interventions in HFASD

Outcome research on the effect of psychosocial and pharmacological interventions for people with HFASD has been relatively recent. A limited number of studies have investigated the efficacy of specific treatment approaches and, not surprisingly, no single methodology or intervention strategy has been identified as effective for all individuals with HFASD [53].

### 4.1. Pharmacological Interventions

While several studies document high rates of prescription medication use in children with ASD [54–58], very little is known about medication use in adolescence and adulthood. There is even less research on medication prevalence and pharmacological interventions for adults and teens with HFASD, despite high rates of psychiatric comorbidity in this population. In one of the only studies to examine these issues, Martin and colleagues [59] studied psychotropic drug use in a high-functioning (IQ > 70) sample of children, adolescents, and adults with autism, AS, and PDD-NOS. Across all diagnostic categories, 55% of participants reported taking psychotropic drugs, and 29% were taking 2 or more medications simultaneously. The most commonly prescribed medications were antidepressants (32%), followed by stimulants (20%) and neuroleptics (17%). Similar findings are described in a more recent study of psychotropic medication use in children with HFASD where 33% of the sample reported taking psychotropic drugs, with stimulant (25%), antidepressant (10%), and neuroleptic (6%) medications most commonly prescribed [60]. More general findings for studies of children, adolescents, and adults with ASD suggest that greater age, lower adaptive skills, lower social competence, higher levels of challenging behaviour, and living away from home are all associated with an increased likelihood of medication use [54, 55, 57, 58, 61, 62]. What these studies fail to describe in detail is the prescriber role and level of care received. Specifically, who is the prescriber and how well is medication monitored over time? The only study to look at medication profiles over time found that as adolescents with ASD aged, medications were only increased but never decreased [61]. Given high rates of psychotropic medication use observed in this population, combined with evidence of lack of psychiatric access, there remain significant concerns around prescribing and monitoring practices.

For studies evaluating the outcome of psychotropic medication use, it is important to ensure that validated, treatment-sensitive, rating scales, and side effects measures are utilized [63]. This is particularly pertinent for adults with HFASD, who can be quite sensitive to medication side effects and resistant in complying with medication regimens. It is also important to note that many medications have adverse or paradoxical side effects [63, 64] and that very few evidence-based approaches to psychopharmacologic management exist for this population [63, 65]. Training of future mental health clinicians must ensure that psychotropic prescription and monitoring for this population are included within curriculum alongside any other psychiatric disorder [33, 34].

### 4.2. Social Skills Interventions

A recent systematic review of research on behavioral interventions for young adults with HFASD identified 20 studies aimed at improving adaptive skills for this population [66]. Social interaction skills were most commonly targeted, followed by practical academic skills, vocational skills, and domestic skills. Improvements were noted in 19 of the 20 studies, and procedures involving visual cues, video modeling, self-reinforcement, contingencies, and corrective feedback showed greatest improvement. In another review of social skills training groups for youth with HFASD [67], most studies of social skills groups involving cognitive-behavioural or parent intervention components demonstrated success. Despite this, there are relatively few social skill interventions in place for individuals with HFASD. Several interventions have been developed with the aim of improving social appropriateness, social interest, and conversation skills, but results remain inconsistent [68–75]. Successful approaches have included those targeting social skill building, social skill training [42, 75–81], computer simulation [71, 82], and peer training [72]. The issue with these findings is a lack of consistent outcome measures, small sample sizes, and the limited number of programs studied, making comparisons between programs difficult and the significance of findings challenging to interpret. More rigorous, objective study of effective interventions and outcomes for this population is critical as individuals with HFASD transition into adulthood and the possibility of new vocational and residential environments.

### 4.3. Supported Employment

Very few vocational opportunities and supports exist for adults with ASD despite the fact that there are a number of well-informed, evidence-based vocational models (e.g., supported employment, job coach approaches [83–88]). Although adults with HFASD may be deemed more capable than individuals with ASD and an ID, organizational, sensory, and social deficits continue to impede successful and meaningful employment opportunities for this population.

Supported employment is a vocational rehabilitation system with demonstrated success in helping individuals with HFASD find and maintain employment [83, 84, 87]. For example, results of a two-year supported employment project for adults with HFASD reported significantly longer terms of employment and higher job levels and salaries, compared to individuals not in a supported program [89]. Additionally, in a more recent study involving an employment service for individuals with HFASD, results found that 68% of individuals involved with the service had found employment [85]. Some of the common features of successful supported employment programs include job matching, individualized, strengths-based job supports, communication supports, family supports, fostering self-determination and independence, autism awareness training, and strong relationships between employers, job coaches, and individuals with ASD [90, 91]. Obstacles to successful employment include completing job applications, acclimating to new job routines, communication, and navigating social interactions with coworkers and supervisors [91].

Findings suggest that supported employment systems may be beneficial for some individuals with ASD; however, barriers still exist from the perspective of employers. At the community level, employers must be engaged and incentivized to hire individuals with ASD through evidence that demonstrates the benefits of hiring individuals with autism to both employer and employee. This is particularly important in the context of knowledge that individuals who receive supported employment are hired faster, earn higher disposable incomes, and have lower individual allowances or disability support payments compared to individuals with disabilities who do not receive supported employment [92]. Given the existent foci of vocational rehabilitation within mental health services [93–97], inclusive of models like supportive employment, the request for similar models for individuals with HFASD suggests an opportunity for the mental health sector to take a leadership role in supporting the vocational needs of individuals with HFASD. Some of these efforts are already underway in the not-for-profit sector, such as the Specialist People Foundation (Specialisterne), but similar efforts are required in the mental health sector on a much broader scale. There is also a parallel need for more research on supported employment approaches and outcomes in this population as individuals with HFASD transition into adulthood.

### 4.4. Cognitive Behaviour Therapy (CBT) and Mindfulness-Based Therapy (MBT)

The effectiveness of cognitive behaviour therapy (CBT) in addressing anxiety, anger, and social skills deficits in youth with HFASD is well documented in the literature [98–105]. There are fewer studies, however, which have examined the effectiveness of CBT in adults with HFASD. Although no large trials have been published on CBT and adults with HFASD, case study and case series reports suggest that CBT is a promising treatment for depression and anxiety in this population [49, 106, 107]. Results of a recent mindfulness-based therapy (MBT) randomized control trial suggest that this may also represent a promising treatment approach. Significant reductions in depression, anxiety, and rumination were observed in a sample of adults with HFASD who received MBT [108]. While further research is needed in this area, particularly large-scale studies which compare the effectiveness of CBT or MBT versus other treatment options (e.g., medication, counseling, etc.), it is important to note that many mental health clinicians already have the skills to effectively deliver these therapies. Another important next step is to look at what has been successfully employed in community mental health settings and adapting these effective, evidence-based psychiatric, employment, and academic interventions to include individuals with HFASD.

### 4.5. Supports for Families

Parents and other family members of individuals with ASD, because of the complexity of their mental health problems, as well as deficits in social and communicative functioning, frequently take on the role of coordinating the individual's healthcare well into adulthood [109]. It is not surprising then that caring for an individual with ASD is associated with high levels of caregiver burden [110, 111]. Despite this, there is a dearth of literature on interventions and supports for parents and siblings of individuals with ASD, particularly in adulthood. Of the few studies that have been conducted to date, results highlight the need to involve family in the individual's overall treatment plan and the need to support and address the mental health and parenting needs of those supporting and caring for individuals with ASD [112, 113]. Further research is needed to identify what supports are most effective for families of adolescents and adults with HFASD.

## 5. Conclusions

Adolescents and adults with HFASD represent a complex and underserved population. Of the studies completed to date, findings suggest that this subgroup of adolescents and adults faces a multitude of psychiatric and psychosocial issues, alongside significant challenges in accessing services. Social skills deficits for individuals with HFASD persist into adulthood, and adults appear to be at heightened risk for developing depression, low self-esteem, and anxiety. Despite this, very few studies have examined treatment patterns and interventions (pharmacological and psychosocial) for adolescents and adults with HFASD. Evidence is beginning to emerge for interventions targeting this population, including CBT, MBT, and SST, but further large-scale studies which compare the effectiveness of, for example, CBT or MBT versus other treatment options (e.g., medication, counseling, etc.) are required and the need for mental health clinicians trained to apply these techniques is now.

In developing interventions for these individuals, programs must consider what adolescents and adults want. For example, adolescents and adults with HFASD may be more interested in interventions targeting vocational opportunities than interventions targeting social skills. Individuals with HFASD and their family members must be viewed as valuable contributors and fully engaged in this process. Further, researchers must look at issues of service cost and efficiency when evaluating the impact of interventions [15].

The large majority of adults and adolescents with HFASD live at home with their families and, of those employed, most obtain jobs in low level, poorly paid, sheltered or supported employment. It is not surprising therefore that the parents of these teens and adults report a number of challenges and concerns related to future independence and availability of appropriate services and supports.

## 6. Recommendations

Together, findings point to a number of important practice recommendations. First, developmental disability agencies or agencies supporting individuals with ASD must partner with community mental health agencies to help train, mentor, and build capacity to care for this population across the lifespan. It is important to note that many clinicians working within community mental health agencies already have the skills to effectively deliver this care, but programs either preclude their ability to do so or they lack the confidence to work with this population. Second, there is a critical need for community mental health agencies to review their exclusion criteria to include persons with ASD. For example, agencies providing care for persons with mood or anxiety disorders should not exclude individuals on the basis of a diagnosis of ASD. Community mental health agencies have the resources and expertise in mental health, along with the programs to care for individuals with mental health issues (e.g., vocational programs, counseling, and therapies), but will need guidance from developmental disability agencies to successfully adapt these programs for adults with HFASD. Third, developmental disability agencies must reevaluate their inclusion criteria to include persons with HFASD, regardless of IQ, and across the lifespan. Organizations must work together, combining expertise in ASD from developmental disability agencies with knowledge and resources from community mental health agencies. Fourth, there is a need to study and identify programs and supports that are most effective in both school and community settings. To do this will require a full continuum of mental health services including counseling, vocational support, inpatient services, and outpatient services. It will also require a network of experienced clinicians and community partners. Many of these efforts are already underway in pediatric settings; however, these same efforts are required in adolescent and adult mental health services. Finally, there is a need to prepare and equip older youth with HFASD for the transition to adult services. A number of key recommendations and principles can be gleaned from the broader ID education/vocational literature [114] and from studies of individuals with ID transitioning from the pediatric to the adult medical system [115, 116], many of which could be tailored to persons with HFASD. For example, the use of meaningful transition tools (e.g., Transition Planning Inventory [117]; Good 2 Go Readiness Checklists https://www.sickkids.ca/Good2Go/What-we-do/Readiness-checklists/33789-G2G%20Transition%20Readiness%20Checklist%20for%20Patients%20-%20v.%20Nov%202010.pdf) and the importance of transition workers, protocols, and policies [118, 119] are required.

## References

[1] Autism Ontario FORGOTTEN: Ontario Adults with Autism and Adults with Aspergers.

[2] Canadian Institutes of Health Research (2007). *Report on the National Autism Research Symposium*.

[3] Mental Health Commission of Canada (2012). *Changing Directions, Changing Lives: The Mental Health Strategy for Canada*.

[4] National Institute of Mental Health (2007). *Autism Spectrum Disorders (Pervasive Developmental Disorders)*.

[5] Charman T, Clare P (2004). *Mapping Autism Research: Identifying UK Priorities for the Future*.

[6] National Autistic Society (2002). *Taking Responsibility: Good Practice Guidelines—Services for Adults with Asperger Syndrome*.

[7] Royal College of Psychiatrists (2006). Psychiatric services for adolescents and adults with Asperger Syndrome and other autism spectrum disorders. *Council Report CR*.

[8] Belfer ML (2008). Child and adolescent mental disorders: the magnitude of the problem across the globe. *Journal of Child Psychology and Psychiatry and Allied Disciplines*.

[9] Rahman A, Harrington R, Mubbashar M, Gater R (2000). Annotation: developing child mental health services in developing countries. *Journal of Child Psychology and Psychiatry and Allied Disciplines*.

[10] Autism and Developmental Disabilities Monitoring Network Surveillance Year 2010 Principal Investigators (2010). Prevalence of autism spectrum disorder among children aged 8 years—autism and developmental disabilities monitoring network, 11 sites, United States, 2010. *MMWR Surveillance Summaries*.

[11] World Health Organization (1992). *International Classification of Diseases, Tenth Revisions, Criteria for Research*.

[12] American Psychiatric Association (2000). *Diagnostic and Statistical Manual of Mental Disorders*.

[13] American Psychiatric Association (2013). *Diagnostic and Statistical Manual of Mental Disorders*.

[14] Howlin P, Moss P (2012). Adults with autism spectrum disorders. *Canadian Journal of Psychiatry*.

[15] Shattuck PT, Roux AM, Hudson LE, Taylor JL, Maenner MJ, Trani J-F (2012). Services for adults with an autism spectrum disorder. *Canadian Journal of Psychiatry*.

[16] Barnard LE, Harvey V, Prior A, Potter D (2001). *Ignored or Ineligible? The Reality for Adults with Autistic Spectrum Disorders*.

[17] Hofvander B, Delorme R, Chaste P (2009). Psychiatric and psychosocial problems in adults with normal-intelligence autism spectrum disorders. *BMC Psychiatry*.

[18] Mohiuddin S, Bobak S, Gih D, Ghaziuddin M, Matson JL, Sturmey P (2011). Autism spectrum disorders: comorbid psychopathology and treatment. *International Handbook of Autism and Pervasive Developmental Disorders*.

[19] Moseley DS, Tonge BJ, Brereton AV, Einfeld SL (2011). Psychiatric comorbidity in adolescents and young adults with autism. *Journal of Mental Health Research in Intellectual Disabilities*.

[20] Simonoff E, Pickles A, Charman T, Chandler S, Loucas T, Baird G (2008). Psychiatric disorders in children with autism spectrum disorders: prevalence, comorbidity, and associated factors in a population-derived sample. *Journal of the American Academy of Child and Adolescent Psychiatry*.

[21] Skokauskas N, Gallagher L (2012). Mental health aspects of autistic spectrum disorders in children. *Journal of Intellectual Disability Research*.

[22] Solomon M, Miller M, Taylor SL, Hinshaw SP, Carter CS (2012). Autism symptoms and internalizing psychopathology in girls and boys with autism spectrum disorders. *Journal of Autism and Developmental Disorders*.

[23] van Elst LT, Pick M, Biscaldi M, Fangmeier T, Riedel A (2013). High-functioning autism spectrum disorder as a basic disorder in adult psychiatry and psychotherapy: Psychopathological presentation, clinical relevance and therapeutic concepts. *European Archives of Psychiatry and Clinical Neuroscience*.

[24] Worley JA, Matson JL (2011). Psychiatric symptoms in children diagnosed with an Autism spectrum disorder: an examination of gender differences. *Research in Autism Spectrum Disorders*.

[25] Hurtig T, Kuusikko S, Mattila M-L (2009). Multi-informant reports of psychiatric symptoms among high-functioning adolescents with Asperger syndrome or autism. *Autism*.

[26] Lugnegård T, Hallerbäck MU, Gillberg C (2011). Psychiatric comorbidity in young adults with a clinical diagnosis of Asperger syndrome. *Research in Developmental Disabilities*.

[27] Mattila M-L, Hurtig T, Haapsamo H (2010). Comorbid psychiatric disorders associated with asperger syndrome/high-functioning autism: a community- and clinic-based study. *Journal of Autism and Developmental Disorders*.

[28] Munesue T, Ono Y, Mutoh K, Shimoda K, Nakatani H, Kikuchi M (2008). High prevalence of bipolar disorder comorbidity in adolescents and young adults with high-functioning autism spectrum disorder: a preliminary study of 44 outpatients. *Journal of Affective Disorders*.

[29] Russell AJ, Mataix-Cols D, Anson M, Murphy DGM (2005). Obsessions and compulsions in Asperger syndrome and high-functioning autism. *The British Journal of Psychiatry*.

[30] Allik H, Larsson J-O, Smedje H (2006). Health-related quality of life in parents of school-age children with Asperger syndrome or high-functioning autism. *Health and Quality of Life Outcomes*.

[31] Tonge BJ, Brereton AV, Gray KM, Einfeld SL (1999). Behavioural and emotional disturbance in high-functioning autism and Asperger syndrome. *Autism*.

[32] Ghaziuddin M, Greden J (1998). Depression in children with autism/pervasive developmental disorders: a case-control family history study. *Journal of Autism and Developmental Disorders*.

[33] Bellini S (2004). Social skills deficits and anxiety in high-functioning adolescents with autism spectrum disorders. *Focus on Autism and other Developmental Disorders*.

[34] Ghaziuddin M (2005). *Mental Health Aspects of Autism and Asperger Syndrome*.

[35] Mukaddes NM, Hergner S, Tanidir C (2010). Psychiatric disorders in individuals with high-functioning autism and Asperger's disorder: similarities and differences. *The World Journal of Biological Psychiatry*.

[36] Raja M, Azzoni A (2008). Comorbidity of Asperger's syndrome and bipolar disorder. *Clinical Practice and Epidemiology in Mental Health*.

[37] Howlin P (2000). Outcome in adult life for more able individuals with autism or Asperger syndrome. *Autism*.

[38] Fleischer AS (2012). Support to students with Asperger syndrome in higher education—the perspectives of three relatives and three coordinators. *International Journal of Rehabilitation Research*.

[39] Rutherford MD, Baron-Cohen S, Wheelwright S (2002). Reading the mind in the voice: a study with normal adults and adults with Asperger syndrome and high functioning autism. *Journal of Autism and Developmental Disorders*.

[40] Tantam D (2003). The challenge of adolescents and adults with Asperger syndrome. *Child and Adolescent Psychiatric Clinics of North America*.

[41] Kasari C, Rotheram-Fuller E, Locke J, Gulsrud A (2012). Making the connection: randomized controlled trial of social skills at school for children with autism spectrum disorders. *Journal of Child Psychology and Psychiatry and Allied Disciplines*.

[42] Tse J, Strulovitch J, Tagalakis V, Meng L, Fombonne E (2007). Social skills training for adolescents with Asperger syndrome and high-functioning autism. *Journal of Autism and Developmental Disorders*.

[43] Baron-Cohen S, Wheelwright S (2003). The friendship questionnaire: an investigation of adults with Asperger syndrome or high-functioning autism, and normal sex differences. *Journal of Autism and Developmental Disorders*.

[44] Howlin P, Goode S, Hutton J, Rutter M (2004). Adult outcome for children with autism. *Journal of Child Psychology and Psychiatry and Allied Disciplines*.

[45] Howlin P, Volkmar F (2007). The outcome in adult life for people with ASD. *Autism and Pervasive Developmental Disorders*.

[46] Whitehouse AJO, Durkin K, Jaquet E, Ziatas K (2009). Friendship, loneliness and depression in adolescents with Asperger's Syndrome. *Journal of Adolescence*.

[47] Mandell DS, Walrath CM, Manteuffel B, Sgro G, Pinto-Martin J (2005). Characteristics of children with autistic spectrum disorders served in comprehensive community-based mental health settings. *Journal of Autism and Developmental Disorders*.

[48] Narendorf SC, Shattuck PT, Sterzing PR (2011). Mental health service use among adolescents with an autism spectrum disorder. *Psychiatric Services*.

[49] Weiss JA, Lunsky Y (2010). Group cognitive behaviour therapy for adults with Asperger syndrome and anxiety or mood disorder: a case series. *Clinical Psychology and Psychotherapy*.

[50] Ward A, Russell A (2007). Mental health services for adults with autism spectrum disorders. *Advances in Mental Health and Learning Disabilities*.

[51] Weiss J (2008). The family study: parents share their experiences of caring for people with ASD in adolescence and adulthood. *Autism Matters*.

[52] Madar T (2007). My use of mental health related services. *Advances in Mental Health and Learning Disabilities*.

[53] Toth K, King BH (2008). Asperger's syndrome: diagnosis and treatment. *The American Journal of Psychiatry*.

[54] Coury DL, Anagnostou E, Manning-Courtney P (2012). Use of psychotropic medication in children and adolescents with autism spectrum disorders. *Pediatrics*.

[55] Rosenberg RE, Mandell DS, Farmer JE, Law JK, Marvin AR, Law PA (2010). Psychotropic medication use among children with autism spectrum disorders enrolled in a national registry, 2007-2008. *Journal of Autism and Developmental Disorders*.

[56] Mandell DS, Morales KH, Marcus SC, Stahmer AC, Doshi J, Polsky DE (2008). Psychotropic medication use among medicaid-enrolled children with autism spectrum disorders. *Pediatrics*.

[57] Spencer D, Marshall J, Post B (2013). Psychotropic medication use and polypharmacy in children with autism spectrum disorders. *Pediatrics*.

[58] Witwer A, Lecavalier L (2005). Treatment incidence and patterns in children and adolescents with autism spectrum disorders. *Journal of Child and Adolescent Psychopharmacology*.

[59] Martin A, Scahill L, Klin A, Volkmar FR (1999). Higher-functioning pervasive developmental disorders: rates and patterns of psychotropic drug use. *Journal of the American Academy of Child and Adolescent Psychiatry*.

[60] Lopata C, Toomey JA, Fox JD, Thomeer ML, Volker MA, Lee GK (2013). Prevalence and predictors of psychotropic use in children with high-functioning ASDs. *Autism Research and Treatment*.

[61] Esbensen AJ, Greenberg JS, Seltzer MM, Aman MG (2009). A longitudinal investigation of psychotropic and non-psychotropic medication use among adolescents and adults with autism spectrum disorders. *Journal of Autism and Developmental Disorders*.

[62] Lake JK, Balogh R, Lunsky Y (2012). Polypharmacy profiles and predictors among adults with autism spectrum disorders. *Research in Autism Spectrum Disorders*.

[63] Myers SM (2007). The status of pharmacotherapy for autism spectrum disorders. *Expert Opinion on Pharmacotherapy*.

[64] Bradley E, Lofchy J (2005). Learning disability in the accident and emergency department. *Advances in Psychiatric Treatment*.

[65] Warren Z, McPheeters ML, Sathe N, Foss-Feig JH, Glasser A, Veenstra-VanderWeele J (2011). A systematic review of early intensive intervention for autism spectrum disorders. *Pediatrics*.

[66] Palmen A, Didden R, Lang R (2012). A systematic review of behavioral intervention research on adaptive skill building in high-functioning young adults with autism spectrum disorder. *Research in Autism Spectrum Disorders*.

[67] Cappadocia MC, Weiss JA (2011). Review of social skills training groups for youth with Asperger Syndrome and high functioning autism. *Research in Autism Spectrum Disorders*.

[68] Carter C, Meckes L, Pritchard L, Swensen S, Wittman PP, Velde B (2004). The friendship club: an after-school program for children with asperger syndrome. *Family and Community Health*.

[69] Howlin P, Yates P (1999). The potential effectiveness of social skills groups for adults with autism. *Autism*.

[70] Palmen A, Didden R, Arts M (2008). Improving question asking in high-functioning adolescents with autism spectrum disorders: effectiveness of small-group training. *Autism*.

[71] Parsons S, Mitchell P, Leonard A (2004). The use and understanding of virtual environments by adolescents with autistic spectrum disorders. *Journal of Autism and Developmental Disorders*.

[72] Thiemann KS, Goldstein H (2004). Effects of peer training and written text cueing on social communication of school-age children with pervasive developmental disorder. *Journal of Speech, Language, and Hearing Research*.

[73] Schmidt C, Stichter JP, Lierheimer K, McGhee S, O'Connor KV (2011). An initial investigation of the generalization of a school-based social competence intervention for youth with high-functioning autism. *Autism Research and Treatment*.

[74] Turner-Brown LM, Perry TD, Dichter GS, Bodfish JW, Penn DL (2008). Brief report: feasibility of social cognition and interaction training for adults with high functioning autism. *Journal of Autism and Developmental Disorders*.

[75] Webb BJ, Miller SP, Pierce TB, Strawser S, Jones WP (2004). Effects of social skill instruction for high functioning adolescents with autism spectrum disorders. *Focus on Autism and Other Developmental Disabilities*.

[76] Barnhill GP (2002). *Designing Social Skills Interventions for Students with Asperger Syndrome*.

[77] Barry TD, Klinger LG, Lee JM, Palardy N, Gilmore T, Bodin SD (2003). Examining the effectiveness of an outpatient clinic-based social skills group for high-functioning children with autism. *Journal of Autism and Developmental Disorders*.

[78] Konstantareas M (2006). Social skills training in high functioning Autism and Asperger's disorder. *Hellenic Journal of Psychology*.

[79] Rogers J (2000). Interventions that facilitate socialization in children with autism. *Journal of Autism and Developmental Disorders*.

[80] Sloman L, Leef J, Stoddart KP (2004). Child social interaction and parental self-efficacy: evaluating simultaneous groups for children with Asperger syndrome and their parents. *Children, youth and adults with Asperger Syndrome*.

[81] Solomon M, Goodlin-Jones BL, Anders TF (2004). A social adjustment enhancement intervention for high functioning autism, Asperger's syndrome, and pervasive developmental disorder NOS. *Journal of Autism and Developmental Disorders*.

[82] Mitchell P, Parsons S, Leonard A (2007). Using virtual environments for teaching social understanding to 6 adolescents with autistic spectrum disorders. *Journal of Autism and Developmental Disorders*.

[83] Burke RV, Andersen MN, Bowen SL, Howard MR, Allen KD (2010). Evaluation of two instruction methods to increase employment options for young adults with autism spectrum disorders. *Research in Developmental Disabilities*.

[84] García-Villamisar D, Wehman P, Navarro MD (2002). Changes in the quality of autistic people's life that work in supported and sheltered employment. A 5-year follow-up study. *Journal of Vocational Rehabilitation*.

[85] Howlin P, Alcock J, Burkin C (2005). An 8 year follow-up of a specialist supported employment service for high-ability adults with autism or Asperger syndrome. *Autism*.

[86] Ling-Yi, L, Shu-Ning Y, Ya-Tsu Y (2012). A study of activities of daily living and employment in adults with autism spectrum disorders in Taiwan. *International Journal of Rehabilitation Research*.

[87] Lawer L, Brusilovskiy E, Salzer MS, Mandell DS (2009). Use of vocational rehabilitative services among adults with autism. *Journal of Autism and Developmental Disorders*.

[88] Stoddart K, Burke L, King R (2012). *Asperger Syndrome in Adulthood: A Comprehensive Guide for Clinicians*.

[89] Mawhood L, Howlin P (1999). The outcome of a supported employment scheme for high-functioning adults with autism or Asperger syndrome. *Autism*.

[90] Lee GK, Carter EW (2012). Preparing transition-age students with high-functioning autism spectrum disorders for meaningful work. *Psychology in the Schools*.

[91] Müller E, Schuler A, Burton BA, Yates GB (2003). Meeting the vocational support needs of individuals with Asperger Syndrome and other autism spectrum disabilities. *Journal of Vocational Rehabilitation*.

[92] Germundsson P, Gustafsson J, Lind M, Danermark B (2012). Disability and supported employment: impact on employment, income, and allowances. *International Journal of Rehabilitation Research*.

[93] Bond G (2004). Supported employment: evidence for an evidence-based practice. *Psychiatric Rehabilitation Journal*.

[94] Bond GR, Drake RE, Becker DR (2008). An update on randomized controlled trials of evidence-based supported employment. *Psychiatric Rehabilitation Journal*.

[95] Drake RE, Bond GR (2011). IPS support employment: a 20-year update. *American Journal of Psychiatric Rehabilitation*.

[96] Drake RE, Goldman HH, Stephen Leff H (2001). Implementing evidence-based practices in routine mental health service settings. *Psychiatric Services*.

[97] Mueser KT, Clark RE, Haines M (2004). The Hartford study of supported employment for persons with severe mental illness. *Journal of Consulting and Clinical Psychology*.

[98] Chalfant A, Rapee R, Carroll L (2007). Treating anxiety disorders in children with high functioning autism spectrum disorders: a controlled trial. *Journal of Autism and Developmental Disorders*.

[99] Greig A, MacKay T (2005). Asperger's syndrome and cognitive behaviour therapy: new applications for educational psychologists. *Educational & Child Psychology*.

[100] Reaven JA, Blakeley-Smith A, Nichols S, Dasari M, Flanigan E, Hepburn S (2009). Cognitive-behavioral group treatment for anxiety symptoms in children with high-functioning autism spectrum disorders: a pilot study. *Focus on Autism and Other Developmental Disabilities*.

[101] Sofronoff K, Attwood T, Hinton S (2005). A randomised controlled trial of a CBT intervention for anxiety in children with Asperger syndrome. *Journal of Child Psychology and Psychiatry and Allied Disciplines*.

[102] Sze KM, Wood JJ (2007). Cognitive behavioral treatment of comorbid anxiety disorders and social difficulties in children with high-functioning autism: a case report. *Journal of Contemporary Psychotherapy*.

[103] Sze KM, Wood JJ (2008). Enhancing CBT for the treatment of autism spectrum disorders and concurrent anxiety. *Behavioural and Cognitive Psychotherapy*.

[104] White SW, Albano AM, Johnson CR (2010). Development of a cognitive-behavioral intervention program to treat anxiety and social deficits in teens with high-functioning autism. *Clinical Child and Family Psychology Review*.

[105] Wood JJ, Drahota A, Sze K, Har K, Chiu A, Langer DA (2009). Cognitive behavioral therapy for anxiety in children with autism spectrum disorders: a randomized, controlled trial. *Journal of Child Psychology and Psychiatry and Allied Disciplines*.

[106] Cardaciotto L, Herbert JD (2004). Cognitive behavior therapy for social anxiety disorder in the context of Asperger's syndrome: a single-subject report. *Cognitive and Behavioral Practice*.

[107] Hare DJ (1997). The use of cognitive-behavioural therapy with people with Asperger syndrome—a case study. *Autism*.

[108] Spek AA, van Ham NC, Nyklíček I (2013). Mindfulness-based therapy in adults with an autism spectrum disorder: a randomized controlled trial. *Research in Developmental Disabilities*.

[109] Carbone PS, Behl DD, Azor V, Murphy NA (2010). The medical home for children with autism spectrum disorders: parent and pediatrician perspectives. *Journal of Autism and Developmental Disorders*.

[110] Cadman T, Eklund H, Howley D (2012). Caregiver burden as people with autism spectrum disorder and attention-deficit/hyperactivity disorder transition into adolescence and adulthood in the United Kingdom. *Journal of the American Academy of Child and Adolescent Psychiatry*.

[111] Kring SR, Greenberg JS, Seltzer MM (2008). Adolescents and adults with autism with and without co-morbid psychiatric disorders: differences in maternal well-being. *Journal of Mental Health Research in Intellectual Disabilities*.

[112] Singh NN, Lancioni GE, Winton ASW (2006). Mindful parenting decreases aggression, noncompliance, and self-injury in children with autism. *Journal of Emotional and Behavioral Disorders*.

[113] Stoddart KP (1999). Adolescents with Asperger syndrome. Three case studies of individual and family therapy. *Autism*.

[114] Carter E, Trainor A, Sun Y, Owens L (2009). Assessing the transition-related strengths and needs of adolescents with high-incidence disabilities. *Exceptional Children*.

[115] Burke R, Spoerri M, Price A, Cardosi A-M, Flanagan P (2008). Survey of primary care pediatricians on the transition and transfer of adolescents to adult health care. *Clinical Pediatrics*.

[116] Peter NG, Forke CM, Ginsburg KR, Schwarz DF (2009). Transition from pediatric to adult care: internists' perspectives. *Pediatrics*.

[117] Clark GM, Patton JR (1997). *Transition Planning Inventory: Administration and Resource Guide*.

[118] Singh SP (2009). Transition of care from child to adult mental health services: the great divide. *Current Opinion in Psychiatry*.

[119] Singh SP, Paul M, Ford T, Kramer T, Weaver T (2008). Transitions of care from child and adolescent mental health services to adult mental health services (TRACK study): a study of protocols in Greater London. *BMC Health Services Research*.

